# Buprenorphine Oral Lyophilisate for Treatment of Opioid Use Disorder: Pharmacology and Clinical Efficacy

**DOI:** 10.3390/ph19020270

**Published:** 2026-02-05

**Authors:** Michael Soyka, Svenja Bolz

**Affiliations:** 1 Department of Psychiatry and Psychotherapy, Ludwig-Maximilians-Universität München, Nussbaumstraße 7, 80336 Munich, Germany; 2 Ethypharm GmbH, 12529 Schönefeld, Germany; bolz.svenja@ethypharm.com

**Keywords:** opioids, opioid use disorder, buprenorphine lyophilisate, treatment

## Abstract

**Background/Objectives**: Opioid use disorder (OUD) is a chronic relapsing condition associated with elevated mortality and substantial psychiatric and somatic comorbidity. Oral methadone and sublingual and depot buprenorphine are the undisputed gold standard in opioid agonist treatment (OAT). More recently, another oral buprenorphine formulation, buprenorphine lyophilisate (BUP-Lyo), has been introduced into clinical practice, offering potentially faster bioavailability and simplified administration. This review aims to summarize the available clinical and pharmacological data on BUP-Lyo and assess its potential role within current OAT strategies. **Methods**: A targeted Medline search was performed to identify publications reporting pharmacological characteristics, safety, efficacy, and clinical use of BUP-Lyo. Additional information was requested from the manufacturer. Relevant sources were reviewed narratively with a focus on OUD treatment, with particular attention to the pharmacological and clinical profile of the compound. **Results**: Few studies on BUP-Lyo have been published to date. As a rapid-dispersion formulation placed on the tongue, BUP-Lyo provides faster bioavailability and a quicker route of administration compared with conventional sublingual buprenorphine. These properties may reduce the need for post-administration supervision and could lessen risks of misuse or diversion. Available evidence supports its safety, efficacy, and feasibility within routine OAT, and the clinical implications of these characteristics are discussed. **Conclusions**: BUP-Lyo expands the range of available buprenorphine formulations and offers practical advantages through accelerated absorption and simplified administration. While initial data are encouraging, the limited evidence base underscores the need for further longitudinal and post-marketing studies to define its clinical position in the management of OUD.

## 1. Introduction

### 1.1. Epidemiology and Diagnosis of Opioid Use Disorder

ICD-10 [1] as well as the novel ICD-11 follow a categorial concept for substance use disorders and distinguish between harmful use and dependence of opioids or other drugs of abuse. Opioid dependence is defined by a cluster of somatic, psychological, and behavioural symptoms. ICD-10 requires three out of six criteria to meet the diagnosis. This categorial distinction between harmful use and dependence is maintained in ICD-11 [2]. In ICD-11, the number of symptoms required for diagnosis have been reduced from six to three.

The Diagnostic and Statistical Manual of Mental Disorders [3] does not follow a categorial but a dimensional approach. Opioid use disorder is defined by a set of eleven criteria. If the patient meets two or three of these symptoms a mild disorder can be diagnosed, four or five symptoms indicate a moderate, six or more symptoms a severe use disorder. Key symptoms of opioid dependence are the compulsion to seek opioid use, loss of control in opioid consumption, withdrawal symptoms, or negative emotional states when opioid use is not possible.

Opioid use disorder is widely recognized as a chronic relapsing disorder with an overall age-standardized prevalence rate of about 0.5% worldwide [4]. In 2017, worldwide 40.5 million people were dependent on opioids and 120,000 people died from opioid overdose [4,5,6,7]. More than 70,000 overdose deaths were attributed to synthetic opioids in the United States in 2022 [7]. In addition, the economic and social burden of drug use disorders is very significant [6,8,9,10]. The Disability-adjusted Life Years (DALYs) for drug use disorders have increased over the past 30 years in Europe [8,11]. In addition to heroin, a number of other opioids are abused, namely opioid pain killers such as fentanyl or oxycodone, as well as opioid maintenance drugs such as methadone and more recently novel synthetic opioids such as nitazenes [12,13,14,15].

### 1.2. Neurobiology of Opioid Use Disorder—Brief Update

The neurobiological basis of opioid use disorder (OUD) has been studied in detail [2]. In the brain, opioids act via different opioid receptors—mu, kappa, and delta—predominantly located in the periaqueductal grey, locus coeruleus, rostral ventral medulla, and substantia gelatinosa of the dorsal horn of the spinal cord. Opioids mediate pain perception, stress, and mood. Opioid receptors are G-protein coupled receptors and inhibit adenyl cyclases and decrease cyclic adenosine monophosphate levels. Endogenous opioid peptides (beta-endorphin, enkephalin, and dynorphin) bind to the opioid receptors. There are three subtypes of opioid receptors. The mu opioid receptor binds to beta-endorphin and mediates reinforcing actions of opioids, dependence, euphoria, respiratory depression, and miosis. Delta opioid receptors bind to enkephalins and mediate analgesia and gastric motility. Kappa receptors bind to dynorphin and provide analgesia, diuresis, and dysphoria [16,17]. For a review on the genetics and neurobiology of opioid dependence see [18,19,20,21,22]. In brief, there is a close functional interrelationship between opioid receptors and dopamine release in the midbrain. Mu opioid receptors are essential for addiction on opioids [17] and regulate dopamine release in the limbic system (nucleus accumbens), the key pathway for reward and addiction [22,23,24] via GABAergic interneurons.

Medications used for treatment of opioid use disorder shall suppress symptoms of the opioid withdrawal and reduce opioid use and craving for opioids. The withdrawal syndrome lasts up to 4–7 days after the last heroin intake. Symptoms include tremor, sweating or hot and cold flushes, runny nose, anxiety, sleeplessness, nausea and vomiting, diarrhea and lack of appetite, and craving for opioids. The neurobiological basis of the opioid withdrawal syndrome is a noradrenergic hyperactivity in the locus coeruleus [25].

### 1.3. Clinical Course of Opioid Use Disorder

OUD is characterized by an often chronic course with multiple relapses, low abstinence rates on the long run and a significant comorbidity with severe somatic and psychiatric disorders and a high mortality and suicide rate [26]. Comorbid or subsequent psychiatric and physical disorders include hepatitis, HIV, other infections or carcinoma, cardiovascular und respiratory disorders, suicide, and accidents [17,27]. There is an excess all-cause mortality in opioid users [8,28].

### 1.4. Existing Treatments

Key objectives in the treatment of OUD are reduction in opioid use and risk of nonfatal and fatal overdose. Secondary goals are improvement in physical and mental health, increase in retention rates, respectively, adherence to treatment, social functioning, and reduction in criminality, among others. Psychosocial therapies used in OUD are motivational interviewing, contingency management [29], and cognitive behavioural therapy (CBT) [30,31,32,33,34]. They have limited efficacy, especially with respect to long-term abstinence ([35], meta-analysis by Berglund et al. [36] and Dutra et al. [37]). In the latter study moderate effect sizes were found for contingency management, relapse prevention, general CBT and treatments combining CBT, and contingency management in OUD [37].

Two Cochrane analyses [38,39] and other extensive studies [40] examined the efficacy of psychosocial interventions in OUD. In general, psychosocial treatments offered in addition to pharmacological detoxification treatments were effective in terms of completion of treatment, opioid use, participants abstinent at follow-up, and clinical attendance [39]. Psychosocial treatments combined with OAT versus OAT alone did not show benefits for retention, use of opioids, and other parameters (28 trials, 2945 participants). Other results suggest that the combination of psychosocial therapies and opioid agonist treatment (OAT) improved the number of participants abstinent at follow-up [38]. Evidence was best for CBT and contingency management [40].

### 1.5. Pharmacotherapy in Opioid Use Disorders

Today OAT with methadone or buprenorphine is the established first-line treatment worldwide [9,41,42,43,44]. Second-line medications are diamorphine (heroin) for treatment refractory patients, morphine sulfate [44], and eventually hydromorphone [45,46,47,48,49].

Numerous studies have shown that full opioid agonists like methadone are first-line medications in the treatment of OUD [2,9,50]. Methadone (MET) is a full agonist at the mu opioid receptor and all other opioid receptors and also an antagonist at the glutamatergic N-methyl-D-Aspartate (NMDA) receptor. MET has a half-life of about 24–36 h and a nearly 100% bioavailability, usually given as a single daily dose (60–100/120 mg daily). MET suppresses symptoms of opioid withdrawal for about 24 h and reduces craving for opioids and opioid-induced euphoria. MET, like other full opioid agonists, produces dose-dependent analgesia and sedation. There is a risk for respiratory depression when overdosed or other CNS depressants are used.

The other first-line medication for OUD is buprenorphine (BUP) [51], a partial mu opioid receptor (OR) agonist, also an antagonist at the kappa opioid receptor. Due to an extensive first-pass metabolism in the liver, BUP has a rather low bioavailability after oral administration. It is absorbed sublingually and metabolized via CYP3A4 and to a lesser extent CYP2C8 in the liver to the active metabolite norbuprenorphine. Cytochrome P450 (CYP) 3A4 mediates 14-N-dealkyation of BUP to norbuprenorphine, which can undergo further glucuronidation.

Oral/sublingual BUP is available in two forms: BUP only and a BUP/naloxone combination. Naloxone, as a fast-acting opioid antagonist, has a poor sublingual but excellent parenteral bioavailability. Naloxone precipitates opioid withdrawal only when administered intravenously. The combination of BUP and naloxone shall reduce the risk of i.v. use of dissolved tablets and diversion [2].

BUP has a long half-life and binds to opioid receptors for at least 24 h. Initial doses are 2–4 mg/day [10] which can be increased by 2–4 mg/day. BUP in doses > 8 mg was shown to suppress opioid withdrawal for 24 h. BUP has a “ceiling” effect at the opioid receptor on respiratory effects—higher doses of BUP > 24 mg produce no or little increase in opioid effects and respiratory depression. Studies suggest the BUP doses < 8 mg are insufficient to produce opioid receptor blockade and are less effective than higher doses [52,53].

The ceiling effect at the opioid receptor reduces the risk of overdose and respiratory depression compared to methadone [54]. Studies also indicate that retention rates for BUP are equal to somehow lower compared to MET [26,55,56]. For details see [2,26,43].

The typical BUP dosage for maintenance treatment is 8–12/16 mg/day. The Food and Drug Administration (FDA) limits the dose to a maximum of 24 mg/day [43]. As for methadone, higher doses of buprenorphine are more effective than lower doses [57]. A meta-analysis of 21 randomized clinical studies showed that the retention rate for BUP is better at a higher dose (16–32 mg/day) than at a lower one (<16 mg/day) [57]. Favourable retention outcomes for buprenorphine have also been found in studies in primary care settings [58].

Numerous studies and meta-analyses have demonstrated the efficacy of both MET and BUP [59,60], also with respect to all-cause mortality [61]. There is evidence for a lower mortality risk in patients treated with BUP compared to MET [62,63].

In recent years three long-acting depot or implant medications for buprenorphine have been granted approval in many countries [56,64,65].

The induction phase of BUP treatment is important for and predictive of treatment outcome, especially for BUP [57]. A lower dropout rate was found if the dose was increased more rapidly in the induction phase [66].

There are numerous studies comparing the outcomes in BUP and MET patients [26]. A meta-analysis on both medications (Degenhardt et al. 2023 [67]) indicates that the treatment retention is better for MET compared to sublingual BUP with other outcomes showing few statistically significant differences. In contrast, another recent meta-analysis on treatment retention in opioid agonist therapy comparing observational studies (N = 3) and randomized controlled trials (N = 10) found methadone to be equal to buprenorphine [68]. In sum, both medications are efficient medications for treatment of OUD.

BUP causes the typical opioid-related side effects including sedation, constipation, decreased appetite, sweating, and fatigue [69]. Many clinicians and patients feel that BUP compared to MET causes less sedation or is more often associated with a “clearheadedness”. BUP can also cause anxiety, sweating, constipation, headache, insomnia, nausea, dizziness, asthenia, and somnolence, similar to methadone.

## 2. Buprenorphine Lyophilisate (Espranor) as a Therapeutic Approach

In recent years a novel pharmacological buprenorphine compound (buprenorphine oral lyophilisate, BUP-Lyo) was introduced into clinical practice. There are 8 mg and 2 mg oral lyophilisate tablets available. The key characteristics of this drug are described below. This review aims to obtain an overview of published or available data on the clinical and pharmacological data of this compound.

Methods: First, a Medline research was performed for BUP-Lyo (5 hits). Only two of these hits were related to the medication reviewed [70,71]. Another publication was found by google research, not published in a Medline listed journal but a peer-reviewed journal [72]. This database was not small for a standardized PRISMA evaluation. Second, the manufacturer was addressed for further pharmacological data.

### 2.1. Pharmacology

The oral buprenorphine lyophilisate is a rapidly dispersing formulation of BUP [70]. The drug is administered by placing it on the tongue.

#### 2.1.1. Pharmacokinetics (Regulatory Assessment Report [73])

Three bioequivalence studies were conducted, two pilot studies with 2 and 8 mg and one pivotal study with 8 mg, see Table 1, Table 2 and Table 3.

Pilot study 1 was a randomized, open-label, two-way cross-over study using BUP-Lyo 2 mg versus BUP 2 mg sublingual tablets. Six healthy subjects participated. Serum levels were followed for 120 h following dosing. Data indicate comparable serum levels (AUC, see Table 1).

Pilot study 2 was conducted in eight healthy subjects. Again, this was a randomized, open-label, two-way cross-over study. Serum drug levels were followed for 72 h after dosing. Serum levels were comparable in both groups (see Table 2).

Study 3 (pivotal study) also was an open-label, two-way cross-over study in 36 healthy adults. BUP-Lyo 8 mg was compared to 8 mg BUP sublingual. Data are given in Table 3. The test and reference product were not within conventional 90% Cl limits of 80–125% on any of the measured parameters and indicate that BUP-Lyo is more available than the reference product. Data indicate higher plasma concentrations which occurred later. The overall exposure was higher in BUP-Lyo.

#### 2.1.2. Clinical Studies

The most relevant study has been conducted by Strang et al. [70]. Key findings and conclusions are described below.

Strang et al. [70] conducted a randomized (2:1) open-label study with 36 opioid-dependent subjects, including subjects consuming alcohol, cocaine, and benzodiazepines, and compared buprenorphine oral lyophilisate (BUP-Lyo, N = 23) with standard sublingual buprenorphine tablets (N = 13). In a 7-day dose titration period buprenorphine dose was initially 2–4 mg on the first day with an additional 2–4 mg if clinically required. The dose then was increased daily, according to clinical response, up to a maximum dose of 24 mg for BUP-Lyo and 32 mg/day for sublingual buprenorphine (BUP-SL). During a 7-day maintenance period the dosing was fixed and during the extension period (days 15–29), all BUP-Lyo subjects were switched to sublingual buprenorphine, either directly or tapered, over a period of up to 4 days.

There also was a pharmacokinetic sub-study in 11 subjects with multiple blood samples on day 1 of the titration period and days 2 and 7 of the maintenance period, and the last day of the extension period. Buprenorphine and norbuprenorphine concentrations were measured by liquid chromatography mass spectrometry.

Mean outcome measures were retention and adherence to treatment, (“medication hold”) and dose adequacy (Likert scales), and opioid withdrawal symptoms as measured by the Objective Opiate Withdrawal Scale (OOWS) and the Subjective Opiate Withdrawal Scale (SOWS). Other measures were the oral disintegration time and monitoring of respiratory rate and various other biological parameters.

Results indicate a slightly but nonsignificant higher retention for BUP-Lyo compared to BUP-SL across the 28-day study (87% vs. 77%) and at the end of the titration phase (96% vs. 85%) as well as the end of the maintenance period (91% vs. 85%).

In addition, there were no differences concerning intensity of opioid withdrawal symptoms and craving for opioids.

##### Bioavailability

After oromucosal administration peak plasma concentrations (C_max_) of buprenorphine are reached in about 70 min, with a linear relationship between doses of 2–8 mg and C_max_. The mean elimination half-life of buprenorphine is 32 h. In the feces, 70% of the drugs are eliminated.

##### Dosing and Safety

BUP-Lyo was titrated at a mean dose of 10.8 +/− 4.9 mg and BUP-SL at 9.6 +/− 4.3 mg. In the cross-over phase, no dose adjustments for the former BUP-Lyo group were necessary. In terms of safety, there were no differences concerning respiratory depression or adverse events. No deaths occurred and no treatment-emergent adverse events resulted in withdrawal. There were three events of oral hypoesthesia from two BUP-Lyo subjects.

##### Pharmacology

**Figure 1:**
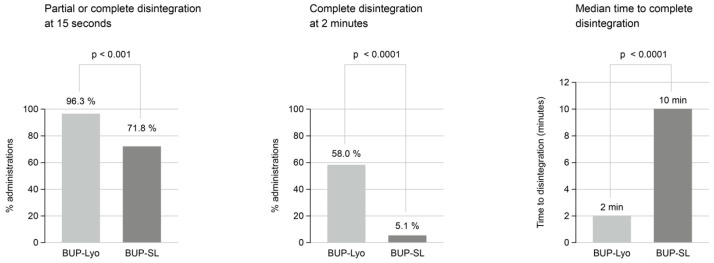
Formulation-specific differences in disintegration time between buprenorphine oral lyophilisate (BUP-Lyo) and buprenorphine sublingual tablets (BUP-SL). Figure created based on data reported in Reference [70]. Oral disintegration time of buprenorphine oral lyophilisate (BUP-Lyo) and conventional sublingual tablets (BUP-SL) was assessed by direct observation at dosing occasions. Two endpoints were recorded: (i) time to disintegration, defined as the time point at which the dosage form could no longer be removed intact, and (ii) time to complete disintegration. The analysis population comprised all subjects who received at least one dose of study medication (BUP-Lyo: n = 23; BUP-SL: n = 13). Data are summarized across all dosing periods. The proportions of administrations achieving partial or complete disintegration within ≤15 s and complete disintegration at 2 min are shown, together with median time to complete disintegration. Group comparisons are presented using descriptive statistics and reported *p*-values, as derived in the original study analyses.

A partial disintegration on the tongue in less than 15 sec was observed in 96.3% of the BUP-Lyo and 71.8% of BUP-SL administrations (*p* < 0.001) (Figure 1). 58.0% of BUP-Lyo and 5.1% of the BUP-SL administrations were completely dissolved at 2 min (*p* < 0.0001). Median time for tablets to completely disintegrate were 2 min for BUP-Lyo and 10 min for BUP-SL (*p* < 0.0001).

##### Pharmacokinetics

Blood samples were available from 11 subjects only (8 BUP-Lyo and 3 BUP-SL). Thus, PK curves were based on a small size. Since subjects were on individualized maintenance doses, PK curves for a dose-adjusted 4 mg dose were examined. In addition, norbuprenorphine PK profiles between BUP-Lyo and BUP-SL were evaluated; BUP-Lyo subjects demonstrated a higher mean (SD) buprenorphine availability (C_max_: 185.8 +/− 88.2% and AUC_0–3 h_: 169.8 +/− 62.0%) relative to BUP-SL. The norbuprenorphine bioavailability was comparable (C_max_:109.6 +/− 42.2% and AUC_0–3 h_: 105.0 +/− 39.4%) relative to BUP-SL.

Additional data for one arm of this study were published by Reed et al. [72]. Subjects in this arm were initially randomized to BUP-Lyo and switched back to standard BUP-SL during an extension period. Patients were reassessed 14 days after switching. Twenty-one of the twenty-three subjects were randomized and receiving BUP-Lyo medication completed the BUP-Lyo maintenance period. One patient was lost to follow-up and twenty subjects entered and completed the switch to BUP-SL. No subjects withdrew due to adverse events. There were no differences in mean maintenance for the BUP-Lyo treatment period (10.8 mg +/− 4.85 mg) compared to the extension period with BUP-SL (10.5 mg +/− 4.98 mg).

Pharmacokinetic data are based on eight patients only, three of them did not have sampling performed at the end of extension. For the remaining five individuals with different dosages, significant increases in mean +/− SD buprenorphine C_max_ and AUC_0–3 h_ were observed with BUP-Lyo relative (%) to BUP-SL (C_max_ 185.8 +/−88.2%, AUC_0–3 h_ 169.8 +/− 62.0%, *p* < 0.001). For norbuprenorphine, the relative % mean +/− SD PK parameter differences were non significantly different when comparing BUP-Lyo and BUP-SL (C_max_ relative 109.6 +/− 42.2%, AUC_0–3h_ 105.0 +/− 39.4%). The authors concluded that the bioavailability with BUP-Lyo is higher that with BUP-SL while norbuprenorphine bioavailability is similar.

Another exploratory analysis from this dataset focused on respiratory depression. Measurements included oximetry scores which were linked to plasma buprenorphine and norbuprenorphine levels [71]. Respiratory depression increased with corresponding exposure levels of buprenorphine and particularly with norbuprenorphine. A lower buprenorphine/norbuprenorphine ratio was predictive of respiratory depression. The mean observed ratio was significantly higher for BUP-Lyo compared to BUP-SL.

#### 2.1.3. Observational Study by Langridge and Bromley [74]

This was a prospective, time, and motion study carried out at two UK prisons. There were two observation periods: one week prior to adoption of buprenorphine oral buprenorphine lyophilisate and one week following the adoption. The primary endpoint was the time required for supervision of buprenorphine administration visits pre- and post-adoption of BUP-Lyo. There were 120 post-administration episodes—50 episodes in the pre-buprenorphine period, 70 in the post BUP-Lyo observation period. The overall OAT administration time per episode was lower in the post BUP-Lyo period (median 2.8, 2.2–3.6 min) compared to the pre-BUP-Lyo period (median 7.3, 5.9–8.2 min) (*p* < 0.001). Other secondary variables included changes in cost to the opioid substitution treatment (OST) service, prescribed dose, and patient and staff experiences associated with the change from tablets to BUP oral lyophilizate. The aspects of OAT supervision, including time to present identification, time to prepare or administer medication, supervision time, and time for checking had dissolved, were all significantly lower in the post-BUP lyophilisate observation period (see Table 1 in [74]). There was no change in prescribed dose of BUP per episode between the pre- and post-BUP oral lyophilisate observation periods (median (IQR) 10.0 (6.5–13.5) mg vs. 10.0 (8.0–14.0) mg, respectively. Semi-structured interviews with patients and staff indicated that buprenorphine oral lyophilizate resulted in less diversion in comparison to other OSTs. However, patients were able to find ways to conceal and divert BUP oral lyophilisate, something that will require further monitoring. BUP-Lyo was well tolerated, seven patients experienced non-serious adverse events (rash, sleep disturbances, sweating, others).

## 3. Discussion

Multiple lines of evidence suggest that the full mu opioid agonist methadone as well as buprenorphine are the undisputed gold standard in the treatment of OUD [2,10]. The safety and efficacy of both drugs have been demonstrated in many studies [2,67]. For the partial opioid agonist buprenorphine, a variety of pharmacological formulations exist, including sublingual buprenorphine with and without the opioid antagonist naloxone and various depot formulations [2,9,10,65]. More recently an alternate buprenorphine formulation, buprenorphine lyophilisate has been introduced into clinical practice and widens the available pharmacological options.

BUP-Lyo is a rapid-dispersion formulation of BUP [70]. The drug is administered by placing it on the tongue. Compared to sublingual formulations of BUP, it disperses more rapidly and has a greater availability. Possible clinical advantages resulting from this are a reduction in post-administration supervision time and diminished risk for misuse and diversion.

There are few studies published so far which indicate that the drug has an increased bioavailability compared to conventional SL buprenorphine. The dosages used are similar and equivalent for BUP-Lyo compared to BUP-SL and the safety profile is similar although the increased bioavailability may have implications for drug dosing [74]. In clinical practice and taking the range of individual drug regimens into account the difference is probably not very significant. The fast absorption of the drug compared to conventional BUP-SL results in an apparently increased bioavailability for BUP-Lyo with increased BUP but not norbuprenorphine bioavailability [70]. Faster absorption of BUP may improve induction and feasibility of buprenorphine treatment. Adequate dosing of buprenorphine is essential for the outcome [53]. It probably reduces the risk for diversion and definitely shortens time needed for delivery of medication [74]. The apparent benefit of BUP-Lyo compared to BUP-SL comes from its faster dissolution time. This means that the patient may spend less time in outpatient facilities or pharmacies when they are consuming BUP-Lyo under supervision [74].

This is of relevance in many treatment settings and may facilitate management of patients in OAT. In an outpatient facility with 100 patients treated on a daily basis, 8 min less time needed for buprenorphine treatment, as indicated by Strang et al. [70], means 800 min less time needed for dispersion of buprenorphine and more time available for clinical or psychosocial management of patients. The possible benefits from this pharmacological profile has to be studied in larger trials.

This is of relevance, especially in prison settings, as shown in the study of Langridge and Bromley [74]. In general, OAT in prison settings has clearly been shown to be effective and reduces mortality rate in opioid-dependent prisoners [75,76,77]. A faster absorption of buprenorphine may also lower the risk of diversion.

Higher plasma levels of BUP may also be beneficial for patients with severe liver disorder/hepatic impairment [78].

Side effects of BUP-Lyo are identical with those of conventional BUP formulations and include QT prolongations with higher doses. The FDA in 2022 issued a warning about severe dental issues linked to buprenorphine medicines dissolved in the mouth (sublingual and buccal tablets/films). Whether this also applies for the faster dissolved BUP-Lyo formulation must be seen. All BUP formulations induce precipitated withdrawal in patients on full opioid agonists. The withdrawal might be heightened of accelerated in BUP-Lyo compared to conventional BUP formulations.

## 4. Limitations

A number of limitations must be addressed. First, the number of published data so far is very limited and long-term studies are missing. The studies published so far have limited power and the data published on respiratory depression are exploratory. Second, larger trials comparing the clinical effects of BUP-Lyo with conventional sublingual or long-acting buprenorphine formulations (head-to-head comparison) are necessary to elucidate its clinical potential. Third, some of data reviewed have been provided by the manufacturer and have not been published in peer-reviewed journals. Fourth, data from prison studies may be difficult to interpret and a number of confounding factors such as selection bias must be kept in mind. Finally, longitudinal and “real-world” studies are missing, especially addressing the use of BUP-Lyo in different treatment settings.

## 5. Conclusions

In conclusion OAT is the undisputed gold standard in the treatment of OUD [9,10,41,42,43,79,80]. A number of different short and long-acting buprenorphine formulations are available now. BUP-Lyo is a safe and efficient opioid suitable and approved for OAT. The essential advantage is a much faster absorption compared to conventional sublingual formulations which may facilitate clinical management of patients, especially in supervised settings. More longitudinal and post-marketing “real world” studies are warranted to define its place in the management of patients with OUD. Especially a head-to-head comparison (non-inferiority RCT comparing BUP-Lyo to standard BUP with adequate power) would be helpful to elucidate its clinical efficacy.

## Figures and Tables

**Table 1:** Pharmacokinetic results of pilot bioavailability study of buprenorphine 2 mg oral lyophilisate compared with sublingual buprenorphine 2 mg (n = 6).

Parameter	Test	Reference	Ratio T/R%	90% CI
C_max_ (ng/mL)	2.22	1.79	124.44	95.76–161.71
AUC_0–t_	11.48	10.75	106.80	41.37–275.74
AUC_0–inf_	15.81	16.11	98.15	42.73–225.43

Table reproduced with permission from Public Assessment Report Espranor 2 mg and 8 mg oral lyophilisate [73]. AUC_0–inf_: Area Under the Curve from zero to infinity; AUC_0–t_: Area Under the Curve from time zero to the last measurable concentration; CI: Confidence interval; C_max_: Peak plasma concentration; ng/mL: Nanograms per milliliter; R: Reference; T: Test.

**Table 2:** Pharmacokinetic results of pilot bioavailability study of buprenorphine 8 mg oral lyophilisate compared with sublingual buprenorphine 8 mg (n = 8).

Parameter	Test	Reference	Ratio T/R%	90% CI
C_max_ (ng/mL)	6.11	7.53	81.17	58.96–111.73
AUC_0–t_	51.72	53.83	96.09	71.49–129.14
AUC_0–inf_	59.74	60.97	97.99	71.08–135.10

Table reproduced with permission from Public Assessment Report Espranor 2 mg and 8 mg oral lyophilisate [73]. AUC_0–inf_: Area Under the Curve from zero to infinity; AUC_0–t_: Area Under the Curve from time zero to the last measurable concentration; CI: Confidence interval; C_max_: Peak plasma concentration; ng/mL: Nanograms per milliliter; R: Reference; T: Test.

**Table 3:** Pharmacokinetic results of pivotal bioavailability study of buprenorphine 8 mg oral lyophilisate compared with sublingual buprenorphine 8 mg (n = 36).

Parameter	Test	Reference	Ratio T/R%	90% CI
C_max_ (ng/mL)	5.77	4.45	129.51	118.94–141.02
AUC_0–t_	26.71	19.42	137.50	122.15–154.78
AUC_0–inf_	30.42	22.14	137.40	121.27–155.67

Table reproduced with permission from Public Assessment Report Espranor 2 mg and 8 mg oral lyophilisate [73]. AUC_0–inf_: Area Under the Curve from zero to infinity; AUC_0–t_: Area Under the Curve from time zero to the last measurable concentration; CI: Confidence interval; C_max_: Peak plasma concentration; ng/mL: Nanograms per milliliter; R: Reference; T: Test.

## Data Availability

No new data were created or analyzed in this study. Data sharing is not applicable to this article.

## Abbreviations

The following abbreviations are used in this manuscript:

AUC	Area Under the Curve
BUP	Buprenorphine
BUP-Lyo	Buprenorphine lyophilisate
BUP-SL	Sublingual buprenorphine
CBT	Cognitive behavioural therapy
CI	Confidence interval
C_max_	Peak plasma concentration
CYP	Cytochrome P450
DALYs	Disability-adjusted Life Years
FDA	Food and Drug Administration
ICD	International Statistical Classification of Diseases and Related Health Problems
IQR	Interquartile Range
MET	Methadone
OAT	Opioid agonist treatment
OOWS	Objective Opiate Withdrawal Scale
OST	Opioid substitution treatment
OUD	Opioid use disorder
SD	Standard deviation
SOWS	Subjective Opiate Withdrawal Scale
